# Hyperexcitability of muscle spindle afferents in jaw‐closing muscles in experimental myalgia: Evidence for large primary afferents involvement in chronic pain

**DOI:** 10.1113/EP090769

**Published:** 2023-12-16

**Authors:** Dar'ya Sas, Fanny Gaudel, Dorly Verdier, Arlette Kolta

**Affiliations:** ^1^ Département de Neurosciences Université de Montréal Montréal Québec Canada; ^2^ Centre Interdisciplinaire de Recherche sur le Cerveau et l'Apprentissage (CIRCA) Montréal Québec Canada; ^3^ Faculté de Médecine Dentaire Université de Montréal Montréal Québec Canada

**Keywords:** astrocytes, chronic muscle pain, ectopic firing, muscle spindle afferent, trigeminal system

## Abstract

The goals of this review are to improve understanding of the aetiology of chronic muscle pain and identify new targets for treatments. Muscle pain is usually associated with trigger points in syndromes such as fibromyalgia and myofascial syndrome, and with small spots associated with spontaneous electrical activity that seems to emanate from fibers inside muscle spindles in EMG studies. These observations, added to the reports that large‐diameter primary afferents, such as those innervating muscle spindles, become hyperexcitable and develop spontaneous ectopic firing in conditions leading to neuropathic pain, suggest that changes in excitability of these afferents might make an important contribution to the development of pathological pain. Here, we review evidence that the muscle spindle afferents (MSAs) of the jaw‐closing muscles become hyperexcitable in a model of chronic orofacial myalgia. In these afferents, as in other large‐diameter primary afferents in dorsal root ganglia, firing emerges from fast membrane potential oscillations that are supported by a persistent sodium current (*I*_NaP_) mediated by Na^+^ channels containing the α‐subunit Na_V_1.6. The current flowing through Na_V_1.6 channels increases when the extracellular Ca^2+^ concentration decreases, and studies have shown that *I*_NaP_‐driven firing is increased by S100β, an astrocytic protein that chelates Ca^2+^ when released in the extracellular space. We review evidence of how astrocytes, which are known to be activated in pain conditions, might, through their regulation of extracellular Ca^2+^, contribute to the generation of ectopic firing in MSAs. To explain how ectopic firing in MSAs might cause pain, we review evidence supporting the hypothesis that cross‐talk between proprioceptive and nociceptive pathways might occur in the periphery, within the spindle capsule.

## Abstract (highlights)

- **What is the topic of this review?**
  Firing in muscle spindle afferents (MSAs) is generated when their muscle is stretched, but sometimes generated elsewhere along the neuron (ectopic firing) in pathological pain. Appearance of ectopic firing coincides with appearance of pain. We examine mechanisms underlying emergence of ectopic firing in MSAs of the jaw‐closing muscles.
- **What advances does it highlight?**
  Firing in MSAs emerges from oscillations supported by a persistent sodium current, which varies with extracellular Ca^2+^, which is, in turn, regulated by astrocytes. We emphasize the potential role of astrocytes in appearance of ectopic firing. We proposed an explanation for how activity in MSAs crosses to nociceptors in the periphery.

## 1 INTRODUCTION

Muscle pain is a perplexing common condition that can, at times, arise from inflammation caused by minor injuries, strain, exercise, fatigue or stress, whereas at other times, pain can be completely absent in some muscle diseases associated with important inflammatory histopathological signs (Partanen, 2018). It is usually localized and resolves spontaneously or after simple treatments (Tantanatip & Chang, 2023). However, in 20%−30% of people, acute musculoskeletal pain develops into chronic pain that persists in the absence of inflammation and after tissue healing and can occasionally lead to devastating conditions, such as chronic widespread musculoskeletal pain, chronic fatigue syndrome, and fibromyalgia, which are major causes of disability (Henschke et al., 2008; Itz et al., 2013; Kasch et al., 2008). A classic example is the large proportion (40%−50%) of patients with acute whiplash‐associated disorders who develop a chronic pain condition (Carroll et al., 2008; Kamper et al., 2008). In the orofacial area, chronic musculoskeletal pain of sufficient intensity to impede speech and eating behaviors by affecting jaw muscle motor function and proprioception is reported in 22%−26% of the general population, with 7%−11% experiencing it persistently (Brattberg et al., 1989).

A typical feature of muscle pain syndromes is that pain is not spread uniformly across the muscle but is triggered at some specific points (Partanen, 2018). Trigger points are spots of hardening in the muscle tissue that are painful on compression (Simons et al., 1999). Typically, trigger points are a hallmark of myofascial pain syndrome (Money, 2017; Saxena et al., 2015), but they can also impact fibromyalgia by adding to the level of central sensitization that characterizes this condition (Ge, 2010). Even in EMG studies of healthy muscles, pain caused by the insertion of the EMG needle is elicited in small spots where spontaneous electric activity is encountered, but outside of these spots, there is no pain and no spontaneous activity (Lavelle et al., 2007; Partanen, 2018). Electrical stimulation near these spots is extremely painful even if no visible contraction can be detected, and because the same stimulation elsewhere in the muscle is painless, it has been proposed that pain spots occur when the needle touches an intramuscular nerve terminal (Partanen, 2018). Furthermore, these pain spots have been found often to coincide with increased resistance to the advancement of the needle, which has led to the suggestion that these terminals are located inside muscle spindle capsules (Partanen, 2018). These capsules are lamellated structures of connective tissue that enclose the intrafusal muscle fibers, around which coil the mechanosensitive sensory endings of large‐diameter primary afferents (PAs) of group Ia and II (Banks & Barker, 2004; Hulliger, 1984). In normal conditions, activity in these afferents conveys to the CNS information about muscle length and speed of stretch, whereas pain sensation is typically conveyed by small‐diameter PAs (groups III and IV), termed nociceptors (S. A. Armstrong & Herr, 2023).

Pain that persists in the absence of noxious stimuli and after tissue healing is considered pathological and often results from central or peripheral changes in excitability in nociceptors and their associated circuitry (Arendt‐Nielsen et al., 2011; Colloca et al., 2017; Finnerup et al., 2021; Voscopoulos & Lema, 2010). However, several lines of evidence suggest that changes in excitability of large‐diameter PAs might also contribute to appearance of pathological pain (Djouhri et al., 2012; Han et al., 2000; Khan et al., 2002; Y. I. Kim et al., 1998; C. N. Liu et al., 2000; Tal M et al., 2006; Tashima et al., 2018; Zhu & Henry, 2012). Here, we review this evidence and focus on the potential role of changes in excitability in muscle spindle afferents (MSAs) in chronic myalgia, using jaw‐closing muscles as an example. We examine the potential contribution of astrocytes, the most common type of glial cells, to these changes in excitability. Finally, we propose a model explaining how changes in excitability in proprioceptive pathways might lead to activation of nociceptive pathways translating into pain sensation.

## 2 EVIDENCE THAT LARGE‐DIAMETER PRIMARY AFFERENTS CONTRIBUTE TO PAIN SENSATION

Firing in PAs is usually generated in the periphery when their receptor in muscle, tendon or skin is activated by a stimulus. When generated elsewhere along the neuron, it is termed ectopic (LaMotte, 2007). In humans, after nerve injury, and in several animal neuropathic pain models, an increase in excitability of large‐diameter PAs leading to spontaneous ectopic firing coincides precisely with pain onset (Han et al., 2000; Khan et al., 2002; C. N. Liu et al., 2000; Tal M et al., 2006). In these conditions, vibration or light mechanical stimuli that activate only large‐diameter PAs can produce pain (Colloca et al., 2017; Zhu & Henry, 2012). More importantly, in mice in which transmission from large‐diameter PAs is left intact, but in which transmission specifically from nociceptors is prevented genetically (conditional VGluT2 knockouts), mechanical hypersensitivity that is normally observed after nerve injury develops and persists despite the fact that they have altered acute nociceptive responses and lose injury‐induced heat hyperalgesia (Scherrer et al., 2010).

Even in milder pain conditions not involving nerve injury, such as delayed onset muscle soreness, which results from excessive exercise or exercises with eccentric contractions and which is often used as a model of muscle pain in humans, there is evidence that specific stimulation of MSAs exacerbates the pain (Barlas et al., 2000; Weerakkody et al., 2001). To reproduce the pH changes observed in muscles after exercise or inflammation in humans or induced by increased levels of muscle lactate and pyruvate observed in chronic myalgia patients (Gerdle et al., 2008, 2010; Hood et al., 1988; Issberner et al., 1996; Reeh & Steen, 1996; Sjogaard et al., 2010), Sluka et al. (2001) developed an animal model using two injections of acidic saline spaced 2–5 days apart. Such injections made unilaterally in the gastrocnemius muscle produced bilateral hyperalgesia, which persisted for 4–5 weeks, even if they caused minimal damage and produced only a small reduction in the pH (to 6.0) that lasted for ∼10 min.

To obtain direct measures of MSA excitability in chronic myalgia conditions, Lund et al. (2010) took advantage of the trigeminal system innervating the orofacial region, where most PAs have their somata in the trigeminal ganglion, while those of MSAs and about half of the periodontal receptors, which are both large‐diameter PAs, are centrally located in the trigeminal mesencephalic nucleus (NVmes), making them easily accessible for identified recordings in brainstem slice preparations. They adapted Sluka's model to the jaw‐closing (masseter) muscles of rats and showed that two ipsilateral injections of acidic saline (20 μL, pH 4.0) caused a bilateral increase in mechanical sensitivity (allodynia) of the masseters that lasted for ∼5 weeks. This increase in sensitivity was accompanied by increased expression of the activity marker c‐Fos and several electrophysiological changes in NVmes cells.

In in vitro preparations from control animals, NVmes cells are usually silent, but if the cells are depolarized they display fast membrane potential oscillations, from which firing emerges. The rising phase of the oscillations depends on a persistent Na^+^ current (*I*_NaP_), while a low‐threshold, 4‐aminopyridine‐sensitive outward current, is probably responsible for the repolarizing phase (Pedroarena et al., 1999; Wu et al., 2001). Normally, the amplitude of the oscillations rises with increasing levels of membrane depolarization. A resurgent Na^+^ current (*I*_res_) activated during the oscillations sometimes leads to rhythmic bursting, but otherwise, it is difficult to maintain firing in these afferents because of a marked outward rectification with depolarization (Del Negro & Chandler, 1997; Verdier et al., 2004). However, in the muscle pain model, a greater proportion of cells displayed spontaneous or sustained firing upon depolarization in the acid‐treated groups versus control animals despite a surprising hyperpolarizing shift of the membrane potential which, counterintuitively, resulted in increased excitability. This is because the thresholds for firing, bursting, and fast membrane oscillations were also shifted to hyperpolarized potentials. The amplitude of the oscillations recorded from the acid‐treated neurons was significantly greater than the amplitude of those recorded from control animals at similar membrane potentials. These differences persisted for ≥35 days but returned to control values by 42 days after the injections, the time at which responses to mechanical stimuli also returned to control levels (Lund et al., 2010).

Large dorsal root ganglion (DRG) neurons have similar membrane potential oscillations, and in various neuropathic pain models involving injury or inflammation of nerves or of DRGs, the amplitude of these oscillations increases and leads to spontaneous somatic ectopic firing (Amir et al., 1999; Ke et al., 2012; C. N. Liu et al., 2000; Xie et al., 2012). Ectopic firing is believed to be a major contributor to chronic neuropathic pain (Devor & Seltzer, 1999), because onset of allodynia or hyperalgesia coincides with a sudden increase in the proportion of large DRG neurons expressing spontaneous firing and large membrane potential oscillations (C. N. Liu et al., 2000). In humans, injections of low doses of local anaesthetic into the DRG reduce phantom limb pain (Vaso et al., 2014). In animal models, blocking spontaneous activity by perfusing the damaged nerve with TTX prevents development of chronic pain, and perfusion of inflamed DRGs with riluzole, which blocks *I*_NaP_, also blocks spontaneous activity and bursting in Aαβ fibre afferents and reduces pain behavior for ≥3 weeks after application (Xie et al., 2012, 2005). Thus, there seems to be a clear link between changes in excitability of large‐diameter PAs and some forms of chronic pain.

## 3 CHANGES IN EXPRESSION OF ION CHANNELS UNDERLYING HYPEREXCITABILITY OF LARGE‐DIAMETER PRIMARY AFFERENTS

Many ion channels are differentially regulated in chronic pain states (reviewed by Alles & Smith, 2021; Bennett et al., 2019), but Na_V_1.3 and Na_V_1.6 are of particular interest for appearance of ectopic firing (X. Liu et al., 1999; Xie et al., 2015). Na_V_1.3 is an embryonic type of channel, whose expression is normally suppressed in adults. It is highly re‐expressed in all injured sensory neurons, but preferentially in medium‐ and large‐diameter DRG neurons (Abe et al., 2002; Black et al., 1999; Dib‐Hajj et al., 1996; C. H. Kim et al., 2001; Waxman et al., 1994). This overexpression of Na_V_1.3 occurs rapidly (within 24 hours of injury) and lasts as long as ectopic discharge and mechanical allodynia are observed (days to weeks) (H. S. Kim & Chung, 1992; C. N. Liu et al., 2000, X. Liu et al., 1999). In humans, Na_V_1.3 is also upregulated in biopsies from trigeminal neuralgia patients (Siqueira et al., 2009).

The second channel of interest, Na_V_1.6, has also been linked to several neuropathic pain states (L. Chen et al., 2020; Henry et al., 2007; Ren et al., 2012). In NVmes cells and in DRG cells, repetitive firing and excitatory persistent and resurgent currents have been shown to rely on *I*_NaP_ and, in particular, on the Na_V_1.6 sodium channel isoform (Cummins et al., 2005; Xing et al., 2014). Spontaneously active bursting cells in inflamed DRGs express higher levels of Na_V_1.6 immunoreactivity, and local knockdown of Na_V_1.6 by injection of small interfering RNA into the DRG at the time of inflammation blocks mechanical pain behaviors and abnormal spontaneous activity in large myelinated PAs, with little effect on unmyelinated PAs in the radicular pain model, the spinal nerve ligation model and the chronic constriction of the sciatic nerve model (Xie et al., 2013, 2015). Furthermore, specific knockdown of Na_V_1.6 in small‐diameter PAs expressing Na_V_1.8 does not affect acute, inflammatory, or neuropathic pain behaviors, further supporting a role for large‐diameter PAs in chronic pain states (L. Chen et al., 2018). Expression of Na_V_1.6 is significantly increased in human skin biopsies taken from patients with complex regional pain syndrome and post‐herpetic neuralgia, and most interestingly, a recent study reported a gain‐of‐function mutation of this subunit in a patient with trigeminal neuralgia, adding to evidence from animal studies to support a role for this channel in pain (Dib‐Hajj et al., 1996; Sittl et al., 2012; Tanaka et al., 2016; Xie et al., 2013, 2015; Zhao et al., 2008).

In all cases, the kinetics and stability of Na_V_ channels are regulated by Ca^2+^, which interacts directly with extracellular moieties and amino acid residues lining the pore of the channel, or indirectly, via its interaction with calmodulin, which modifies the gating behaviors of the channel, or calpain, which cleaves Na_V_ channel α‐subunit Na_V_1.6 (C. M. Armstrong & Cota, 1990; C. Brocard et al., 2016; Santarelli et al., 2007; Sarhan et al., 2012). Decreases of the extracellular Ca^2+^ concentration ([Ca^2+^]_o_) are able to shift the activation threshold of *I*_NaP_ and its half‐activation voltage (by ∼1 mV, every 0.1 mM) towards more hyperpolarized potentials (F. Brocard et al., 2013; Li & Hatton, 1996; Morquette et al., 2015; Su et al., 2001). Thus, even small variations in [Ca^2+^]_o_ can have an important effect on the firing of a neuron by decreasing the threshold for *I*_NaP_ activation and increasing its amplitude, perhaps explaining the increased excitability associated with the hyperpolarizing membrane potential shift observed in the muscle pain model above. Interestingly, Morquette et al. (2015) have shown, in a different trigeminal nucleus, that astrocytes, the most common type of glial cells, exert a powerful effect on *I*_NaP_ by releasing a calcium‐binding protein, S100β, which modulates [Ca^2+^]_o_. On the basis of their findings, Ryczko et al. (2021) went on to show that photostimulation of astrocytes expressing channelrhodopsin (ChR2; a light‐sensitive cationic channel) in glial fibrillary acidic protein (GFAP)‐ChR2 mice causes reductions in [Ca^2+^]_o_ that are sufficient to elicit Na_V_1.6‐driven firing in cortical layer 5 pyramidal neurons, which is prevented by infusion of an anti‐S100β antibody. Taken together, this evidence suggests that astrocytes can alter neuronal firing by releasing a protein, S100β, that potentiates *I*_NaP_ by decreasing [Ca^2+^]_o_.

**FIGURE 1:**
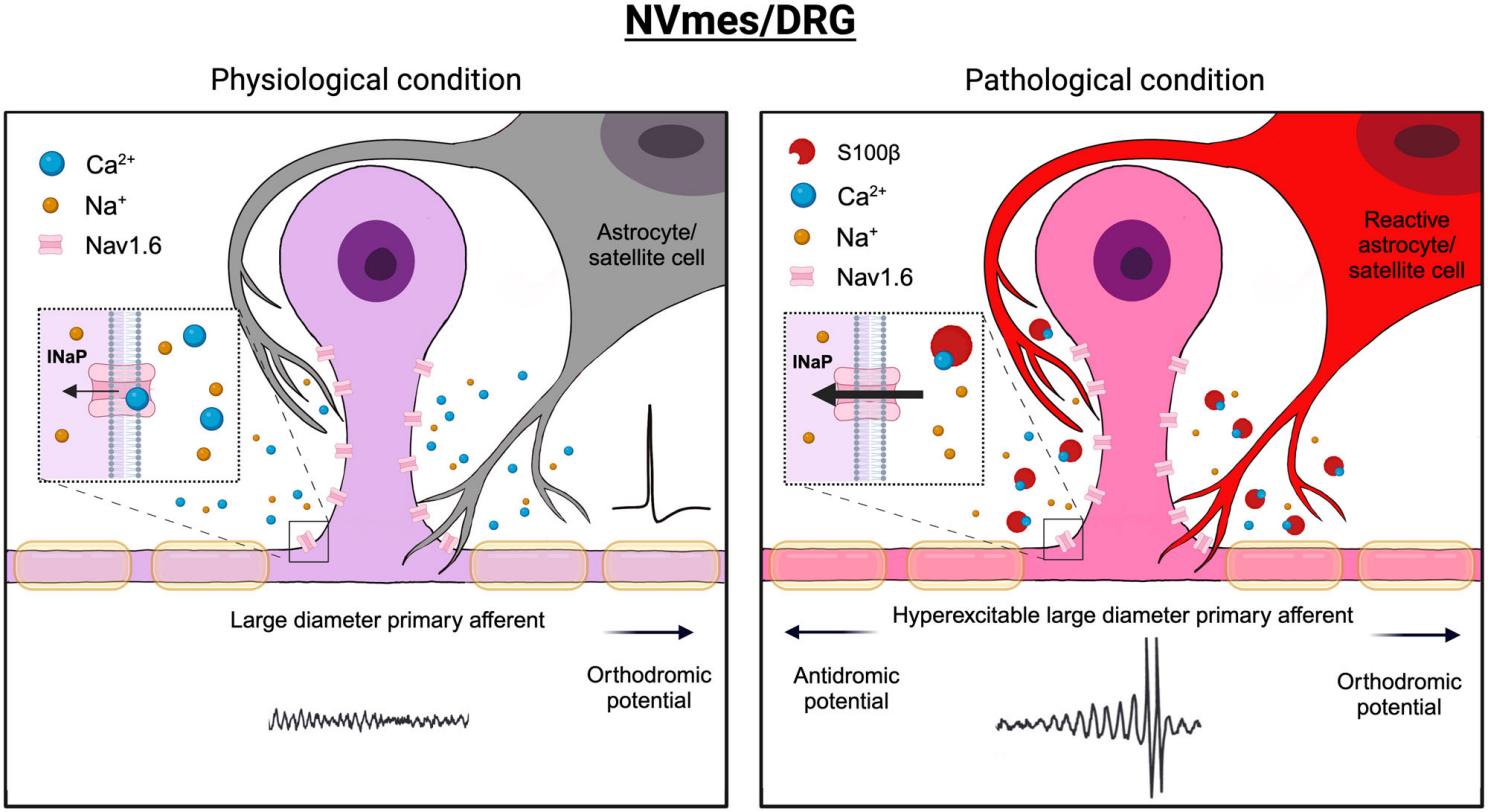
Schematic representation synthesizing the proposed mechanisms underlying the appearance of ectopic firing in large‐diameter PAs of NVmes and DRG neurons. Left: In physiological conditions, higher levels of extracellular Ca^2+^ limit activity in the cells by occluding the Na^+^ channels (Na_V_1.6) mediating *I*_NaP_ that supports membrane potential oscillations from which firing normally emerges. Right: In pathological states, reactive astrocytes (perhaps activated initially by firing of large‐diameter PAs sensing acidosis) release S100β, which chelates extracellular Ca^2+^ and enhances *I*_NaP_, causing an increase in the amplitude of the fast membrane oscillations and emergence of ectopic firing. Abbreviations: DRG, dorsal root ganglion; *I*_NaP_, persistent sodium current; NVmes, trigeminal mesencephalic nucleus; PA, primary afferent.

**FIGURE 2:**
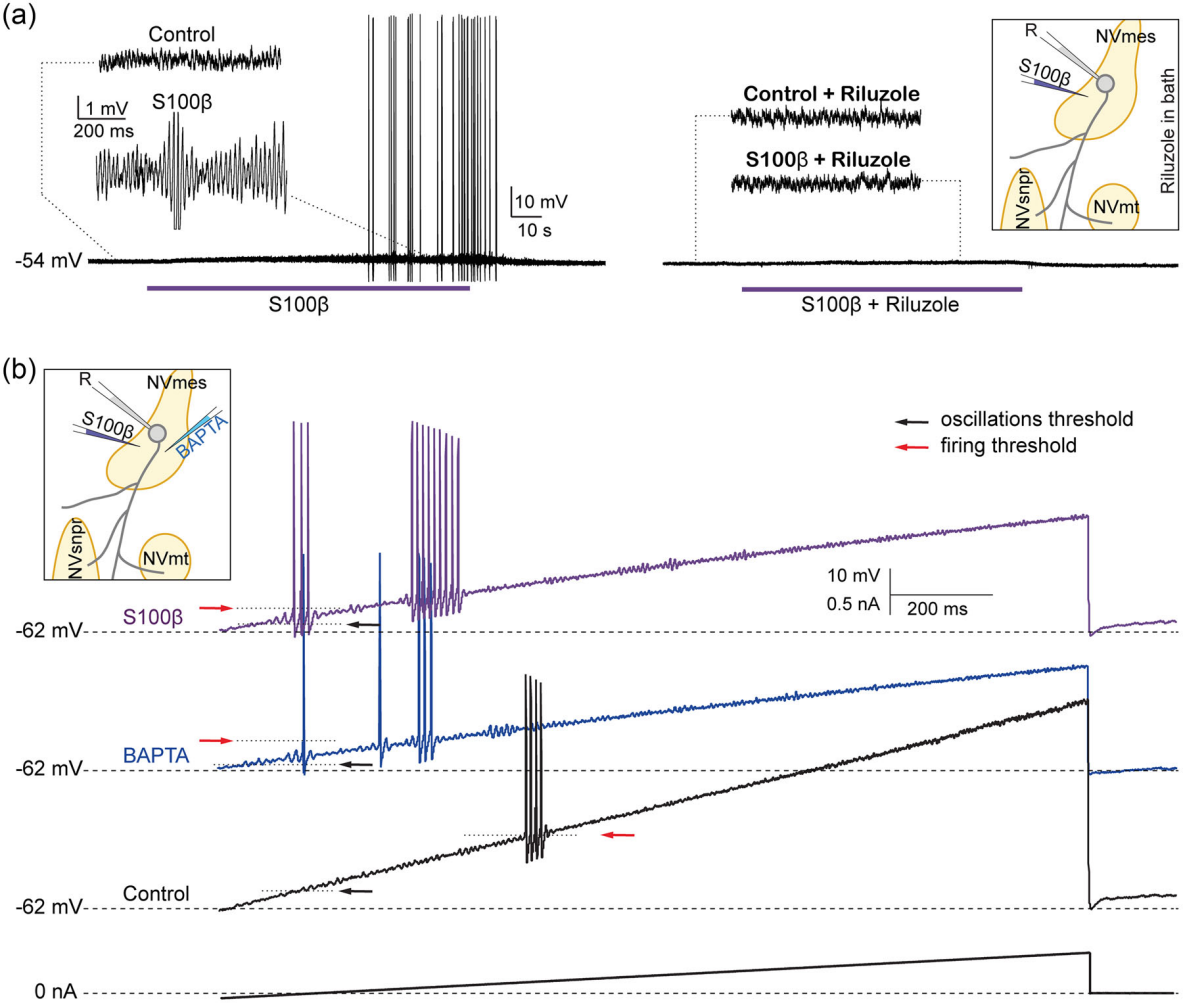
Effect of S100β and BAPTA on oscillations and firing properties of NVmes cells. (a) S100β locally applied on an NVmes cell increases the amplitude of the oscillations and induces firing (left), which is abolished by riluzole (right). (b) Both BAPTA and S100β decrease the threshold for firing (red arrows) and for oscillations (black arrows) in NVmes neurons subjected to a ramp protocol. Insets: Schematic representation of the experimental set‐up. Abbreviation: NVmes, trigeminal mesencephalic nucleus.

**FIGURE 3:**
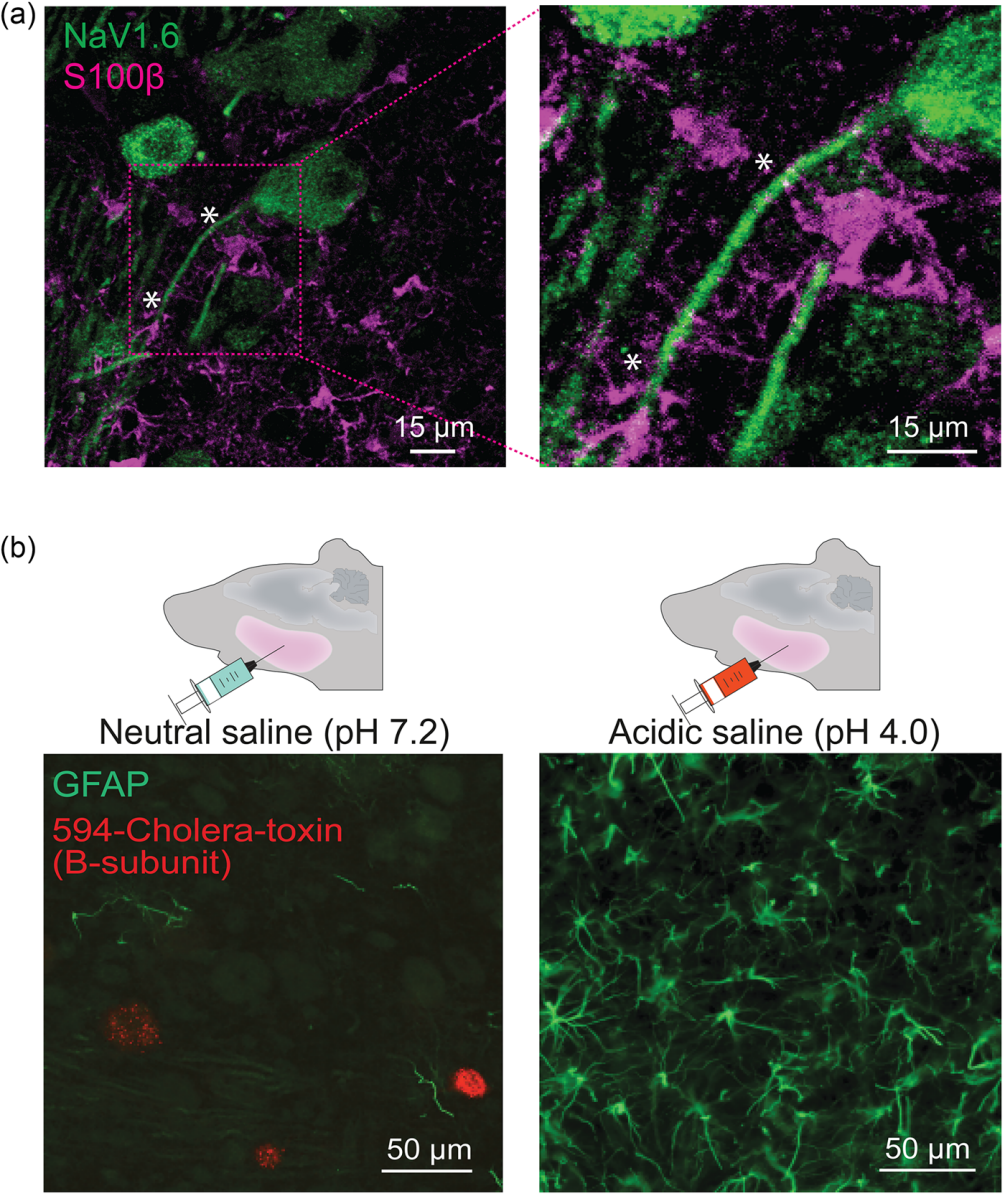
Proximity of astrocytic processes to NVmes axons and their activation in the acidic saline masseteric myalgia model. (a) S100β‐immunoreactive (pink) astrocytes have processes closely apposed (*) to NVmes somata and axons that are immunoreactive to Na_V_1.6 (green). (b) Expression of GFAP (green) in the NVmes area of rats from the control and pain groups at 7 days after the second neutral (left) or acidic (right) saline injection. Somata of NVmes neurons (red) are retrogradely labelled by intramuscular injections of fluorescent cholera toxin β‐subunit. Abbreviations: GFAP, glial fibrillary acidic protein; NVmes, trigeminal mesencephalic nucleus.

These observations raise the possibility that the hyperexcitability of NVmes neurons in the pain model results from an enhancement of *I*_NaP_ caused by a decrease in [Ca^2+^]_o_ following release of S100β from activated astrocytes (Figure 1). Unpublished (except in abstract form) results from Gaudel et al. (2022) show that local applications of S100β near NVmes neurons recorded in a rat brainstem slice preparation increase the amplitude of their oscillations and lead to firing (Figure 2a, left) in previously silent neurons, which is blocked with riluzole (Figure 2a, right), an *I*_NaP_ blocker. S100β, in addition to BAPTA, a Ca^2+^ chelator that reproduces the effects of S100β on [Ca^2+^]_o_, also lowers the firing and oscillation thresholds (Figure 2b). Interestingly, astrocytic processes immunoreactive to S100β were also seen closely apposed onto sections of the axon initial segment of NVmes neurons enriched in Na_V_1.6, as shown in Figure 3a. Thus, experimentally lowering [Ca^2+^]_o_ with local applications of BAPTA or S100β near the axon initial segment of NVmes neurons elicits *I*_NaP_‐dependent firing. This effect could be produced physiologically by release of S100β from astrocytic processes apposed to the axon initial segment.

## 4 POTENTIAL ROLE FOR GLIAL CELLS IN PAIN AND IN MODULATION OF EXCITABILITY OF LARGE‐DIAMETER PRIMARY AFFERENTS

A large body of evidence indicates that glial cells, including satellite cells in DRGs and the trigeminal ganglion, and in microglia and astrocytes in the CNS, play important roles in chronic pain states (Chiang et al., 2011, 2012; Ohara et al., 2009; C. I. Svensson & Brodin, 2010). Several studies have shown that chronic pain is correlated with the appearance of reactive astrocytes, as indicated by increased expression of GFAP (Hansen & Malcangio, 2013; Zhuang et al., 2006). In models of orofacial inflammatory or neuropathic pain, both astroglia and microglia are activated very rapidly (within 20 minutes) in association with central sensitization in the subnucleus caudalis of the spinal trigeminal tract, which is considered functionally equivalent to the medullary dorsal horn (Chiang et al., 2011, 2012; Yeo et al., 2001). Similar increases of GFAP expression were observed in the vicinity of NVmes neurons of rats up to 9 days after injections of acidic saline into their masseters (Figure 3b; unpublished results from Gaudel et al., 2022). The mechanisms underlying this activation are unknown, but in two neuropathic pain models where microglia and satellite glial cells in the DRG are activated, perfusion of a Na^+^ channel blocker (TTX) into the DRG to reduce activity of spontaneously active sensory neurons also prevents glial activation, indicating that glial activation might result initially from an increase in activity of PAs (Xie et al., 2009). Reactive astrocytes, in contrast, release more gliotransmitters, raising the possibility that hyperexcitability of large, myelinated PAs results from increased release of S100β, as illustrated in Figure 1 (and see Agulhon et al., 2012). This is supported by the facts that S100β mRNA and protein are increased in the spinal cord after peripheral inflammation and nerve injury (Tanga et al., 2006) and that S100β knockout mice have decreased mechanical allodynia after nerve injury, whereas mice in which S100β is overexpressed have increased mechanical allodynia (Tanga et al., 2006).

## 5 HOW DO HYPEREXCITED LARGE‐DIAMETER PRIMARY AFFERENTS ACTIVATE NOCICEPTIVE PATHWAYS?

**FIGURE 4:**
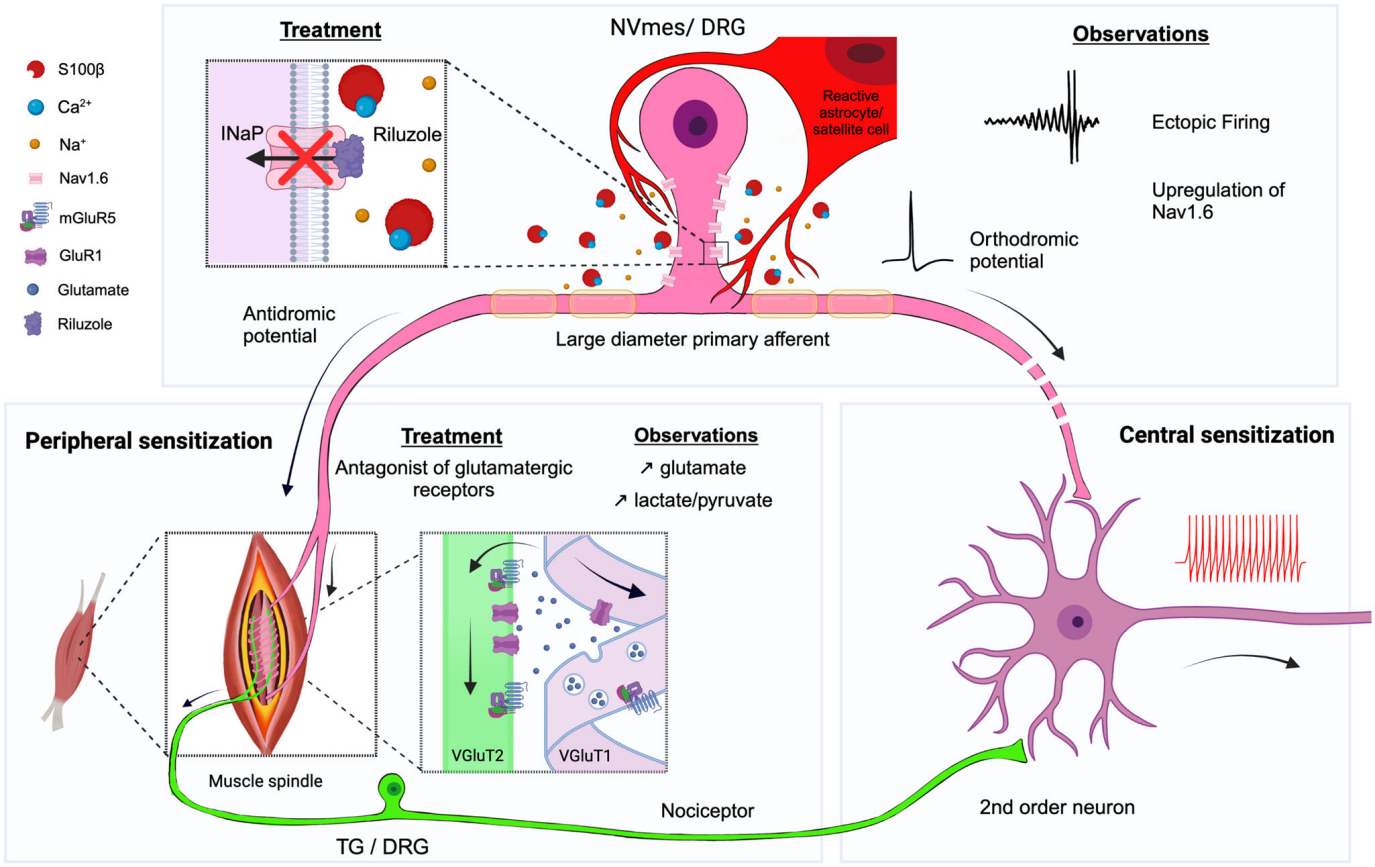
Schematic summary of mechanisms thought to underlie peripheral sensitization within spindle capsules, with indications of all locations where therapeutic interventions have been shown to have analgesic effects in humans. Top: Blockade of *I*_NaP_ with riluzole (left) prevents ectopic firing in NVmes or the DRG (see Figure 1 for mechanisms underlying the appearance of the ectopic firing) and produces analgesia. Bottom left: Unless prevented, the ectopic firing travels antidromically to the peripheral endings of large‐diameter VGluT1‐positive PAs and induces glutamate release within the spindle capsule (using vesicular release), thereby activating nociceptors (which normally express VGluT2) through ionotropic and metabotropic glutamate receptors and contributing to the observed allodynia. Intramuscular injections of ionotropic and metabotropic glutamate receptor antagonists prevent activation of nociceptors and induction of allodynia. Bottom right: Chronic activation of nociceptors (having their soma in the TG or the DRG) and/or central endings of large‐diameter PAs leads to chronic activation of second‐order neurons and central sensitization. Abbreviations: DRG, dorsal root ganglion; *I*_NaP_, persistent sodium current; NVmes, trigeminal mesencephalic nucleus; PA, primary afferent; TG, trigeminal ganglion.

Several central mechanisms might lead to cross‐talk between large‐diameter PAs and nociceptive pathways. Lund & colleagues (2010) postulated that antidromic propagation of ectopic action potentials could cause glutamate release at peripheral endings and be a potential mechanism of activation of nociceptive fibers if they have endings nearby, within the spindle capsule (Figure 4, bottom left).

The possibility that antidromic discharges cause release of a chemical in the periphery was raised by Catton (1961) and Habgood (1950). Glutamate is the major neurotransmitter released by the central terminals of MSAs and thus, the primary candidate for release by their peripheral endings. Glutamate concentrations within the affected muscles increase in chronic myalgia, during delayed onset muscle soreness and following tissue injury or inflammation (deGroot et al., 2000; Lawand et al., 2000; Tegeder et al., 2002). Interstitial glutamate is also higher within the masseter muscles of patients suffering from myofascial temporomandibular disorders in comparison to healthy subjects (Castrillon et al., 2010). We suggest that this glutamate comes, in part, from MSA terminals. Bewick et al. (2005) showed that MSA endings release and recycle glutamate, which serves mainly to modulate their mechanical sensitivity. Injections of glutamate (or its agonists) into muscles, including the masseter, cause pain in humans (Cairns et al., 2003) and excite small‐diameter afferent fibers in animals (Bhave et al., 2001), presumably by activating glutamatergic receptors on their peripheral endings. Indeed, blocking of glutamate receptors decreases nocifensive behaviors in animal models (Dong et al., 2006; Ro & Capra, 2006; Ro et al., 2007; P. Svensson et al., 2003). Release of glutamate from MSA terminals could activate or sensitize muscle nociceptors if they are close to the release sites. Lund et al. (2010) showed that annulospiral MSA endings contain high levels of the glutamate transporter VGluT1, which is usually associated with glutamate release sites (see their figures 5–7). These endings intersected with fine fibers that expressed known nociceptor markers but did not express tyrosine hydroxylase, indicating that they are not sympathetic efferents. Both annulospiral MSA endings and fine fibers expressed metabotropic (mGluR5) glutamate receptors, and ionotropic receptors (GluR1) were also found in fine fibres innervating blood connective tissues. A recent study by Thompson et al. (2023) also confirmed the presence of mGluR5 glutamate receptors on fine‐caliber axons within the spindle capsules. However, the authors reported that only a GluK2 glutamate receptor subunit could be detected on spindle mechanosensory terminals. The homomeric GluK2 receptor formed by this subunit is an atypical metabotropic glutamate receptor coupled to phospholipase D (PLD‐mGluR), whose blockade or activation abolishes or greatly increases stretch‐evoked firing of MSAs, respectively. In line with studies showing that mGluR agonists and antagonists increase and decrease sensitivity, respectively, in an inflammatory pain model (Bhave et al., 2001) and that ionotropic receptor antagonists reduce glutamate‐induced masseter pain in humans (Cairns et al., 2006), Lund and collaborators found that mixtures of either ionotropic or metabotropic glutamate receptor antagonists given together with the second unilateral injections of acid saline prevented the induction of allodynia on both sides. The blockers were ineffective if given 2 days after the second injection (Figure 4; see figure 8 of Lund et al., 2010).

## 6 PERSPECTIVES AND CLINICAL IMPLICATIONS

Traditionally, research into the mechanisms driving chronic pain has focused predominantly on small‐diameter PAs and on mechanisms of either peripheral or central sensitization leading overall to increased activity in nociceptive pathways. However, strong evidence suggests that central sensitization is maintained dynamically by peripheral inputs because it subsides shortly after cessation of peripheral activity (reviewed by Devor, 2009; Gracely et al., 1992; Koltzenburg et al., 1994). This is supported by the observation that in syndromes of chronic widespread pain or fibromyalgia, attenuation of peripheral inputs alleviates or abolishes allodynia and hyperalgesia in patients (J. J. Chen et al., 2010).

Using the findings obtained from MSAs of the jaw‐closing muscles in a chronic myalgia model, in the present paper, we compile evidence supporting the proposal that initial activity in large‐diameter PAs, initiated or amplified by activation of astrocytes or satellite cells, leads to ectopic firing. Firing in these large‐diameter PAs depends on *I*_NaP_, which is enhanced by decreases of [Ca^2+^]_o_. A central hypothesis of the model proposed here is that astrocytes/satellite cells play a major role in the generation of ectopic firing by releasing S100β, a protein that chelates extracellular Ca^2+^. This ectopic firing can travel orthodromically and perhaps lead to increased firing in nociceptive pathways (central sensitization; Figure 4, bottom right), but here we propose that it also travels antidromically and leads to release of glutamate from large‐diameter PAs in the periphery. This released glutamate would then activate glutamatergic receptors carried by nearby free endings of nociceptors, thus initiating firing in nociceptive pathways (peripheral sensitization; Figure 4, bottom left).

Multiple animal models of pain have been designed to mimic distinct clinical diseases to evaluate the underlying mechanisms and potential treatments. The acid‐induced pain animal model, based on the observation of increased levels of lactate and pyruvate in exercised muscles of healthy subjects or muscles of patients with chronic muscle pain, has given inconsistent results in humans, with stronger algesic effects when using injections of a buffered acidic saline solution (pH 5.2) causing longer‐lasting pH changes, instead of an unbuffered solution (even with a lower pH of 3.3) (Castrillon et al., 2013; Law et al., 2008; Louca et al., 2013; Louca Jounger et al., 2019). Recent work from Chen's group and others (reviewed by Lee & Chen, 2023) has described a variety of proton‐sensing ion channels and receptors expressed in proprioceptors, enabling them to sense acidosis. However, it is possible that the inconsistent effects observed with injections in experimental conditions result from the fact that the proprioceptor terminals are isolated in the spindle capsule, whereas in pathological conditions acidification might be produced by muscular satellite cells located within the capsule, given that their presence has been reported in this location by Maynard and Cooper (1973).

Although it is uncertain whether the animal model used is relevant to human physiology, several observations in humans support the proposed theoretical model. First, as stated above, after nerve injury the onset of pain coincides in time with the appearance of ectopic firing in large‐diameter PAs (Campero et al., 1998). This ectopic firing could result from increased expression of some sodium channel subunits, such as Na_V_1.3 and Na_V_1.6, which are upregulated in biopsies from trigeminal neuralgia patients and in patients with complex regional pain syndrome and post‐herpetic neuralgia (Siqueira et al., 2009; Sittl et al., 2012; Tanaka et al., 2016; Zhao et al., 2008). Na_V_1.6, in particular, is responsible for *I*_NaP_, whose blockade with lignocaine or riluzole reduces phantom limb pain and tactile allodynia (Vaso et al., 2014; Xie et al., 2012, 2005).

Second, interstitial glutamate levels are higher in masseter muscles of patients suffering from temporomandibular disorders and in resting, low‐force exercise, or repetitive work in patients with chronic muscle pain, such as fibromyalgia, chronic shoulder pain, and chronic trapezius pain (Gerdle et al., 2010; Gerdle, Ghafouri et al., 2014; Gerdle, Larsson et al., 2014b; Larsson et al., 2008; Malatji et al., 2017; Sorensen et al., 2018). Moreover, glutamate injected into the human masseter muscle or temporomandibular joint causes pain and/or mechanical sensitivity, and intramuscular injection of glutamate receptor antagonists sometimes relieves the pain (reviewed by J. Liu et al., 2022). These observations support the model according to which ectopic firing in large‐diameter PAs resulting from enhanced *I*_NaP_ leads to antidromic firing and release of glutamate within the muscle in the periphery causing pain, perhaps through activation of nociceptor terminals.

## 7 CONCLUSION AND RECOMMENDATIONS FOR FUTURE RESEARCH

The evidence reported here suggests that neuron–astrocyte interactions can cause ectopic spiking and that these interactions are enhanced in pathological pain conditions. Future work should examine how this abnormal firing can be prevented and/or how to prevent communications from MSA terminals to nociceptor terminals in the periphery. This should help to identify new drug targets, some of which are in the periphery, which is a clear advantage, from a therapeutic perspective, for the millions of people suffering from chronic musculoskeletal pain, which is by far the most common type of pain.

## AUTHOR CONTRIBUTIONS

Dar'ya Sas contributed to writing and illustration of the paper. Fanny Gaudel and Dorly Verdier generated some of the data and figures presented. Arlette Kolta contributed to writing of the paper. All authors read and approved the final version of the manuscript and agree to be accountable for all aspects of the work in ensuring that questions related to the accuracy or integrity of any part of the work are appropriately investigated and resolved. All persons designated as authors qualify for authorship, and all those who qualify for authorship are listed.

## CONFLICT OF INTEREST

None declared.
